# Microparticles in Autoimmunity: Cause or Consequence of Disease?

**DOI:** 10.3389/fimmu.2022.822995

**Published:** 2022-04-20

**Authors:** Nils Rother, Cansu Yanginlar, Elmar Pieterse, Luuk Hilbrands, Johan van der Vlag

**Affiliations:** Department of Nephrology, Radboud Institute for Molecular Life Sciences, Radboud University Medical Center, Nijmegen, Netherlands

**Keywords:** microparticles, autoimmunity, microparticle isolation techniques, cell death, cellular communication

## Abstract

Microparticles (MPs) are small (100 nm – 1 um) extracellular vesicles derived from the plasma membrane of dying or activated cells. MPs are important mediators of intercellular communication, transporting proteins, nucleic acids and lipids from the parent cell to other cells. MPs resemble the state of their parent cells and are easily accessible when released into the blood or urine. MPs also play a role in the pathogenesis of different diseases and are considered as potential biomarkers. MP isolation and characterization is technically challenging and results in different studies are contradictory. Therefore, uniform guidelines to isolate and characterize MPs should be developed. Our understanding of MP biology and how MPs play a role in different pathological mechanisms has greatly advanced in recent years. MPs, especially if derived from apoptotic cells, possess strong immunogenic properties due to the presence of modified proteins and nucleic acids. MPs are often found in patients with autoimmune diseases where MPs for example play a role in the break of immunological tolerance and/or induction of inflammatory conditions. In this review, we describe the main techniques to isolate and characterize MPs, define the characteristics of MPs generated during cell death, illustrate different mechanism of intercellular communication *via* MPs and summarize the role of MPs in pathological mechanisms with a particular focus on autoimmune diseases.

## Introduction to Microparticles

**Figure 1:**
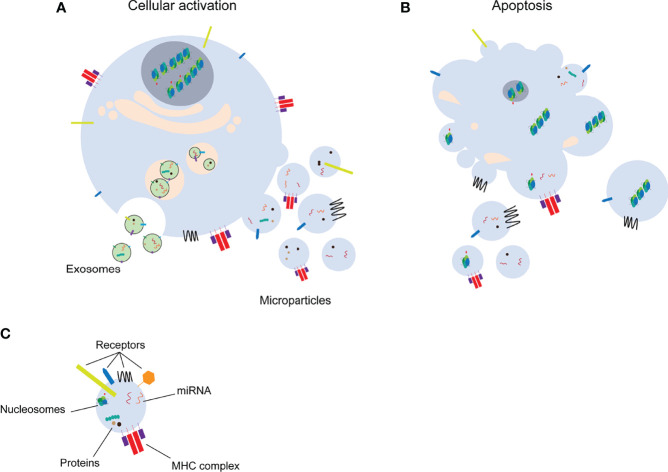
Distinct classes of extracellular vesicles. Upon cell activation **(A)** exosomes or microparticles are formed by exocytosis or membrane shedding, respectively. Microparticles and apoptotic bodies **(B)** are released from apoptotic cells. Extracellular vesicles transfer several molecules to target cells **(C)**. miRNA: microRNA, MHC: major histocompatibility complex.

Microparticles (MPs) are a subclass of extracellular vesicles (EVs) that are a heterogeneous population of vesicles originating from cellular membranes. EVs are important for intercellular communication in a similar manner as cytokines, hormones and neurotransmitters. The release of EVs into the extracellular space occurs mainly *via* two different mechanisms, by cellular activation or during cell death [apoptosis, necroptosis, pyroptosis, reviewed in (1)] (**Figures 1A, B**). The smallest EVs are exosomes. Exosomes are formed by exocytosis of multivesicular bodies with size ranges from 50 nm to 100 nm (2). MPs (100 nm-1 µm) and apoptotic bodies (1 µm-4 µm), on the other hand, are formed by membrane shedding (2) and will be the main focus of this review. Formation of MPs by cell activation or apoptosis, is characterized by calcium influx into the cell. Calcium signaling eventually leads to changes in the cytoskeleton and budding of the cell membrane (3). MPs mediate the communication between different cells by carrying a wide range of molecules including receptors, proteins, RNA species such as miRNAs or other nuclear components (**Figure 1C**). Additionally, MPs can carry mitochondrial proteins, mitochondrial DNA and intact mitochondria [reviewed in (4)]. MPs released into the blood or urine are an easily accessible biomarker to detect differences in cells in response to physiological or pathological changes (5). Circulating MPs used as biomarkers for diagnostic and/or prognostic purposes, may lead to adjustment of therapy based on the individual needs of the patients. However, uncontrolled release of MPs as well as disturbed clearance of MPs, are associated with a wide range of pathologies (including e.g. cardiovascular dysfunction, cancer, autoimmunity) (6–10). In this review, we will focus on experimental techniques to isolate and characterize MPs, the interaction of MPs with immune cells and their pathological roles including induction of different types of cell death and their involvement in autoimmune conditions.

## Experimental Techniques to Isolate, Quantify and Characterize MPs

The technique used to isolate and characterize MPs from blood samples can have a great impact on the concentration and composition of the final MP population (11, 12). The variability in methods makes it challenging to compare results from different studies using divergent methods. To improve reproducibility, it is important to use reliable and standardized methods for isolation, quantification and characterization of MPs.

### Parameters That Influence the Analysis of MPs

Various pre-analytical factors may influence the outcome of MP analysis. Important parameters to consider when examining MPs are: (i) the type of anticoagulant used in the blood sample; (ii) the time between collecting and processing blood samples; (iii) agitation during transport of blood samples; (iv) the protocol of MP isolation (discussed in more detail below); and (v) the storage of MP samples. The choice of anticoagulant can drastically influence MP counts in blood samples, with citrate being the most widely used and advised anticoagulant (13, 14). Time-delay until processing of blood samples and excessive agitation during transport have been shown to increase numbers of MPs, most likely due to activation of cells releasing MPs *ex vivo* after blood withdrawal (13, 15). The duration of storage can have an effect on MP integrity, and therefore, counts. Overall, long term storage tends to decrease MP counts (13, 16). Another factor that might influence the analysis of MPs is whether blood is collected pre- or post-prandial. Blood collected post-prandial is enriched in lipoproteins, which could be co-purified with MPs and interfere with analysis (17, 18).

Other body fluids, such as urine, can also be used for MP isolation. Several strategies have been developed for isolation of MPs from urine (19, 20), as urine derived MPs can be used as fluid biopsy reflecting kidney health (21). Especially, miRNA content of urinary MPs reflects different renal pathologies including IgA nephropathy (22). Various pre-analytical factors, such as time of sample collection, temperature and time of storage, influence the size as well as content of MPs isolated from urine (19).

### Different Methods to Isolate MPs

Different MP isolation protocols may result in variations in number and characteristics of isolated MPs. Differential centrifugation offers an easy and relatively unbiased method of isolating MPs and is therefore the most frequently used method for isolation of MPs from different body fluids or cell culture supernatant. To isolate MPs from blood an initial centrifugation at ~300 x g is performed to obtain platelet-poor plasma. The initial centrifugation is followed by a second centrifugation with higher speeds (15,000 – 20,000 x g) to pellet the MPs. The protocol to isolate MPs from cell culture supernatants usually consists of two centrifugation steps: The first centrifugation step at low speed (800 – 1500 x *g*) aims to eliminate intact cells and/or large particles, whereas the second centrifugation step at high speed (15,000 – 20,000 x *g*) aims to pellet MPs. By using platelet poor plasma from healthy volunteers, Dey-Hazra et al. showed that a first centrifugation step at higher speed (5000 vs. 1500 x *g*) leads to more than 10-fold decrease in final concentration of MPs. However, using this method MPs could easier be separated from background noise in flow cytometry experiments (11, 15). The decreased MP counts obtained with an initial high speed centrifugation step can be attributed to reduced contamination by platelets and/or erythrocytes, thereby avoiding formation of MPs from these sources during the isolation process (16). In addition to affecting the total MP count, the isolation strategy can also have an effect on the relative abundance of MP subspecies derived from different cell types, such as platelets or endothelial cells (16). Differential centrifugation does not yield highly pure MP populations and it can typically contain protein aggregates [such as immune complexes (23)], aggregates of smaller vesicles (24) and lipoproteins (25). However, differential centrifugation is able to recover high amounts of MPs from the starting material compared to more stringent isolation methods, such as affinity isolation, and combinations of other techniques (26).

Other methods to isolate MPs from biological fluids include the use of microfluidic systems. These chip-based isolation systems offer the possibility to isolate MPs from very small sample volumes, which is not possible using differential centrifugation. Microfluidic approaches often use size-based filtration to isolate MPs. However, disadvantages of size-based filtration are clogging of the filter and disintegration of MPs thereby making it challenging to isolate sufficient numbers of MPs for further analysis (27, 28). Antibodies recognizing cell surface markers (such as CD41, to capture platelet-derived MPs or CD63, a tetraspanin often found in MPs) may also be applied to isolate MPs, thereby only enriching a subset of MPs (29–31). Development of an affinity-based MP isolation technique with antibodies requires immobilization of antibody molecules on solid media such as magnetic or agarose beads, and ELISA plates. An elution step with appropriate reagents has to be applied, if bead-MP complexes cannot be directly used for the downstream assays, or in case of coated ELISA plates, which may affect the integrity of isolated MPs (32). Elution-induced effects on MPs can be avoided with a recent method developed by Evander et al., which uses microscale acoustic trapping for non-contact capture of MPs and intact MP recovery (33). Other techniques to isolate MPs include density gradient centrifugation, precipitation, size exclusion chromatography and variations on those techniques [reviewed in (26)]. Differential centrifugation will remain the MP isolation technique of choice in the case of large sample volumes. For small sample volumes microfluidics-mediated methods could serve as alternative if future developments succeed in isolating intact MPs in an unbiased manner and with sufficient numbers for downstream analysis.

While the described isolation techniques result in reliable MP preparations, it is important to keep in mind that none of the described isolation techniques will achieve completely pure MP populations. Contaminants present in MP preparations may include platelets, plasmatic protein aggregates and lipoproteins (25, 34, 35). Therefore, potential contaminations need to be evaluated when analyzing MPs.

### Analysis of MP Numbers and Cellular Origin

**Figure 2:**
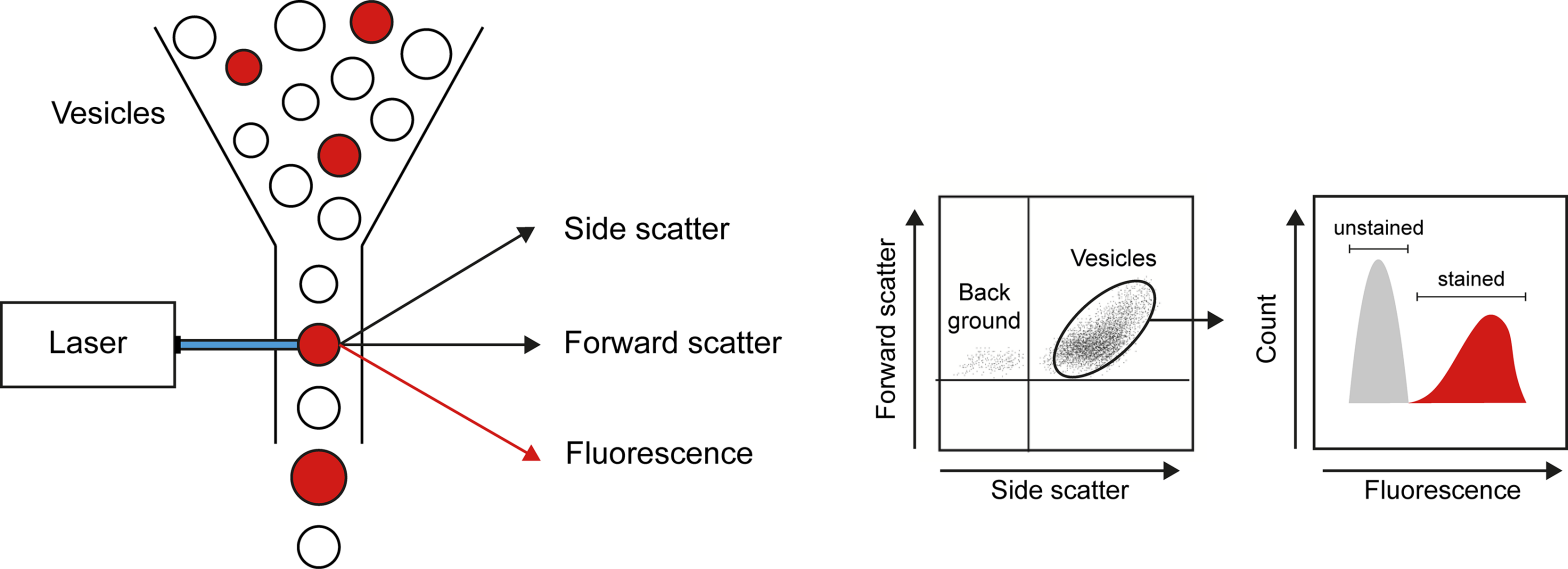
Flow cytometry as a technique to quantify and characterize MPs. By combining data on size (forward scatter), granularity (side scatter), and presence of markers of interest (obtained by use of fluorescently labeled antibodies), flow cytometry allows detection of number, cellular origin, and cargo of MPs.

Flow cytometry is the main technique applied to analyze number and cellular origins of MPs (**Figure 2**). Flow cytometry analysis is restricted by the detection limit of the instrument allowing to analyze only larger MPs. The detection limit of flow cytometers used to be around 0.4 to 0.5 µm, however, state-of-the-art flow cytometers can analyze particles as small as 0.1 μm. Nevertheless, flow cytometry offers a high throughput technique to count and simultaneously analyze different markers to determine the source of MPs. Analysis of the source of MPs relies on different cell-specific markers present on MPs originating from different cell types. However, there is no consensus on cell markers used to identify MPs from e.g. platelets, endothelial cells or leukocytes (36) (**Table 1**), which makes it difficult to compare study outcomes. Assessing cell-specific markers on MPs using flow cytometry can be very challenging as the density of markers on small particles can be very low and therefore difficult to detect. Therefore, highly specialized flow cytometers have been developed for MP characterization [compared in (73)]. Another widely used technique to determine vesicular size and concentration is nanoparticle tracking analysis (NTA). NTA determines the size of vesicles by assessing the Brownian motion of vesicles using laser light scattering microscopy (74). Recent advances in NTA include the possibility to (immune)phenotype MPs by the use of fluorescently labelled antibodies, which in part resembles flow cytometric analysis using fluorescent antibodies (75, 76),

**Table 1:** Markers used to classify the cellular origin of MPs.

Origin	Marker	References
Endothelial cell	CD31 (CD42 neg)*	(37–47)
	CD51**	(38, 47–49)
	CD54**	(38, 39)
	CD62**	(38–40, 44, 45, 47, 50, 51)
	CD105*	(38, 39, 48, 52–54)
	CD144	(16, 45, 54–58)
	CD146	(59–62)
Platelet	CD41	(30, 42, 45, 48, 53, 55, 57, 62–66)
	CD41a	(16, 52, 58, 67–69)
	CD42a	(15, 33, 51, 59, 60)
	CD42b	(39, 44, 46, 56)
	CD61	(48, 61, 66, 70, 71)
	CD142	(15)
Leukocyte	CD45	(37, 44, 52, 55, 57, 59–61, 66, 72)
Monocyte	CD11b	(16)
	CD14	(44, 45, 51, 53, 58, 62, 64–66, 69, 72)
	CD16	(44)
Neutrophil	CD11b	(16)
	CD15	(64, 66, 72)
	CD66b	(37, 45, 58, 62, 66)
T cell	CD3	(37, 53, 62, 64, 69)
cytotoxic	CD8	(69)
helper	CD4	(69, 72)
regulatory	CD25	(62)
B cell	CD19	(37, 53, 69)

*Constitutive, **inducible, without *^/^**: inducibility not indicated

Several groups reported multi-omic analysis of MPs, thereby analyzing the protein and lipid content of MPs (77–80). Kowal and colleagues showed that proteomic analysis of MPs using mass spectrometry led to the identification of proteins defining certain subtypes of vesicles (77). They found proteins uniquely present in bigger MPs (> 150 nm; such as GP96, actinin-4, mitofilin) as well as proteins only present in small MPs (50 – 150 nm; such as syntnein-1, tumor susceptibility 101 (TSG101), a disintegrin and metalloproteinase domain-containing protein 10 (ADAM10), EH domain-containing protein 4 (EHD4) or exosomes. Furthermore, they showed that markers originally used to define exosomes, such as major histocompatibility complex, flotillin and heat shock 70kDa proteins, are also found in larger MPs. In addition to size-based characterization of small MPs and exosomes, tetraspanin-based purification and characterization was applied by Kowal et al. By analyzing the (co)presence and/or (co)absence of CD9, CD63 and CD81, they showed that proteins associated with different stages of the endosomal pathway are enriched in different subgroups of small MPs. Similarly, analysis of CD63+ or CD81+ small MPs and exosomes from B cells revealed different subgroups (81), thereby underscoring the role of tetraspanins in cargo selection and targeting (82). Analysis of MPs by mass spectrometry can also be used to define MP characteristics specific for certain diseases. Ostergaard and colleagues analyzed the proteome of MPs isolated from patients with SLE (78). They found clear differences when comparing MPs from SLE-patients to MPs from healthy controls or to MPs from systemic sclerosis patients. MPs from patients with SLE lack proteins derived from mitochondria, have lower myosin:actin ratios and a high abundance of glycolytic enzymes. These results could lead to further insight into the mechanisms of MP release in SLE or even to more refined diagnostics.

## MPs and Cell Death

Regulated cell death *via* apoptosis is essential for the proper functioning of the human body. Cells undergo apoptosis in all parts of the body and this process is normally tightly regulated. Two forms of apoptosis can be differentiated, namely intrinsic or extrinsic apoptosis [reviewed in (83)]. Intrinsic apoptosis is imitated by perturbations of the intracellular homeostasis, whereas extrinsic apoptosis is mostly triggered by the engagement of plasma membrane receptors. The key step to start the intrinsic apoptosis pathway is the permeabilization of the mitochondrial outer membrane by the BCL2 protein family (84, 85). Subsequently, cytochrome c is released from the mitochondria intermembrane space and activated the caspase cascade ultimately leading to the execution of the cellular remodeling observed during apoptosis (86, 87). Extrinsic apoptosis is triggered by the engagement of so-called death receptors (e.g. Fas-receptor) or by dependence receptors, a family of structurally unrelated membrane receptors that are activated as soon as their substrate concentration falls below a certain threshold level such as DCC (deleted in colorectal carcinoma), p75NTR (p75 neurotrophin receptor) (88–90). Again, both receptor types activate downstream signaling leading to caspase activation. During the process of apoptosis, cells shrink and form multiple blebs on the cell surface. Ultimately, these blebs are released from the parent cell and can enter the circulation as MPs (91, 92). Inherent to the mechanism of apoptosis is the redistribution of cellular components. Next to cytoplasmic proteins, especially nuclear components, such as DNA and histones can be found in apoptotic MPs (37, 93).

Apoptosis involves the tight regulation of major structural changes in the cell to ensure proper packaging of cellular constituents and removal of apoptotic cells by phagocytes in an immune silent manner, prior to the formation of MPs. Even though the exact mechanism of how MPs are formed is not completely understood, several regulators of the cytoskeleton seem to play a role in the formation and the sorting of cellular constituents into MPs. The intracellular increase of calcium concentration observed during MP formation, triggers various processes. First, cytoskeletal rearrangements allows the formation of membrane blebs as precursors of MPs. The calcium dependent protease calpain is involved in the cleavage of structural proteins connecting the plasma membrane with the cytoskeleton (3, 94). Other proteins involved in the remodeling of the cytoskeleton are small GTPases, such as ARF6 and RhoA (95–97). Through the remodeling and resorting of the cytoskeleton, cytoskeletal proteins and GTPases might also be involved in the packaging of specific cargo to the MPs (98). Whereas MPs can contain a myriad of different cargoes representing the entire contents of its parent cell, some studies suggest that MPs can also contain only specific constituents of the cell content (99). Finally, to facilitate release of preformed MPs Rho associated protein kinase (ROCK) and extracellular signal–regulated kinases (ERK) activate myosin light chains to induce contraction of the actin-myosin cytoskeleton (100–102).

Besides rearrangements of the cytoskeleton another prominent change during apoptosis is the reorganization of lipids in the plasma membrane of MPs. The best described change is the reorientation of phosphatidylserine (PS) from the inner lipid layer (inner leaflet) to the outer lipid layer (outer leaflet) of the double lipid layer in cellular membrane. Flippases are responsible for the transport of lipids from the outer leaflet to the inner leaflet and prevent the presence of PS in the outer leaflet of healthy cells. During apoptosis the energy dependent flippase mechanism is shut down and PS reaches the outer leaflet by the unspecific transport of lipids facilitated by scramblases (103). Localization of PS in the outer leaflet of apoptotic cells and ultimately also at the surface of apoptotic MPs serve as an ‘eat me’ signal to specialized phagocytes. Therefore, under normal conditions PS exposure is an efficient mechanism to remove apoptotic cells in a quick and immunologically silent manner. However, when early apoptotic cells are not swiftly cleared, they will proceed to late apoptotic cells, thereby releasing MPs. The relative abundance of PS positive and negative MPs can be affected by certain pathologies. For example, PS negative MPs are predominantly present in systemic lupus erythematosus patients while virtually absent in healthy controls (55, 59). To reliably distinguish MPs from large debris (cell remnants) or protein aggregates, many researchers use staining with Annexin V to identify PS on MPs (11, 16). However, the use of Annexin V as a general MP marker remains a matter of debate. For instance, apoptotic endothelial cell-derived MPs show more binding of Annexin V compared to TNF-α activated endothelial cell-derived MPs (38). Overall, PS at the surface of MPs is an important marker to distinguish between apoptotic and non-apoptotic populations of MPs, but PS is unsuited as a general MP marker.

Interestingly, if apoptotic MPs are not cleared properly, they can in turn induce different forms of cell death when interacting with other cells. MPs generated from Jurkat cells induce apoptosis of macrophages, thereby showing a marked increase in Annexin V staining as well as caspase activation in macrophages (104). Previously, we reported that apoptotic MPs generated from endothelial cells, represent MPs found in SLE patients, both in size and by the presence of specific chromatin modifications. These apoptotic MPs were able to induce dose-dependent neutrophil cell death *via* the formation of neutrophil extracellular traps (NETs) (93). The capability of apoptotic MPs to induce NET formation was found to be dependent on the presence of modified chromatin, especially acetylated histones. Importantly, apoptosis-modified histones within SLE MPs are highly pro-inflammatory, as demonstrated by their potency to activate myeloid and plasmacytoid dendritic cells (37, 93). As macrophages and neutrophils are phagocytotic cells that are specialized in clearing apoptotic material, the observed cell death induction in these cells by apoptotic MPs seems counterintuitive. The cell death of macrophages and neutrophils induced by apoptotic MPs might be explained by the pathologically high concentration of MPs or, in the case of SLE, by the specific pro-inflammatory cargo (e.g. histones) found in SLE-derived MPs.

## Interaction of MPs With Immune Cells

**Figure 3:**
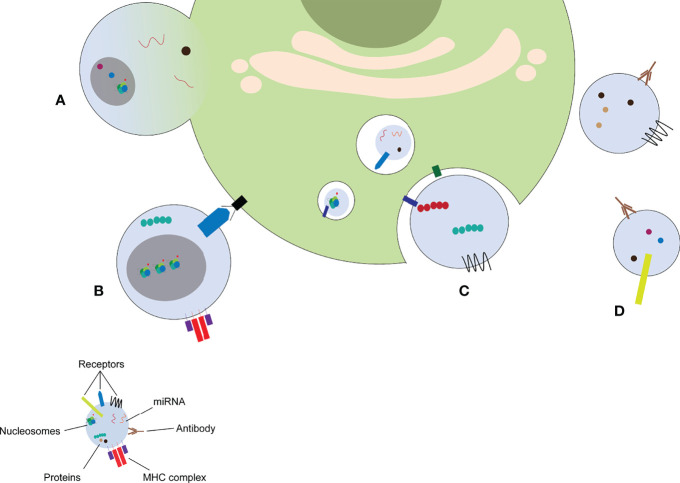
MP induce cellular response *via* different mechanisms. MPs can fuse with the target cell **(A)**, stimulate receptors on the target cell by direct binding **(B)**, release their content into the endosomal compartment **(C)**, or form immune complexes with autoantibodies **(D)**. miRNA: microRNA, MHC: major histocompatibility complex.

MPs released from parent cells can have numerous effects on distant cells. MPs have thus been recognized as mediators of intercellular communication. MPs can exert their effects on other cells *via* different mechanisms (**Figure 3**): MPs can fuse with the target cell (**Figure 3A**), MPs can bind to receptors on the target cell by direct interaction (**Figure 3B**), MPs can be taken up by the target cell *via* endocytosis (**Figure 3C**), ultimately ending in the lysosome where its contents are degraded, and MPs can form immune complexes with antibodies (**Figure 3D**), which can either deposit in the microvasculature or stimulate other cells.

### Fusion of MPs With Target Cells

MPs can fuse with other cells to transfer proteins or other molecules from one cell to another. This transport of MPs has been proposed as a communication strategy between distant cells. MPs originating from activated endothelial cells have been described to carry the intercellular adhesion molecule-1 (ICAM-1) to endothelial cells elsewhere in the body (105). ICAM-1 is then incorporated into the plasma membrane of the target cell, thereby increasing its capability of recruiting immune cells. Besides the transfer of membrane associated receptors like ICAM-1, also the delivery of small non-coding RNAs *via* MPs has been demonstrated. Especially microRNA (miRNA) molecules have been described to be transported between cells (106). miRNAs are capable of regulating the translation of mRNA in the target cell and have been described to be present in MPs released from endothelial cells. These miRNAs can, for example, prevent excessive proliferation of other endothelial cells (107, 108).

### Binding of Antibodies to MPs

MPs have been described to carry different types of immunoglobulins (Igs) (52, 109, 110). Immunoglobulins can be bound to the surface of MPs, but can also recognize components inside MPs (111, 112). MPs found in patients with SLE carry significantly more Ig compared to MPs from healthy controls (110). Additionally, MPs from patients with SLE are covered by depositions of complement proteins C1q and C3, thereby demonstrating that autoantibodies bound to MPs can initiate complement activation (113). A proteomic analysis of MP content in patients with SLE recently confirmed the presence of Ig and complement on MPs (78). MPs could thereby provide a scaffold for immune complex formation in autoimmune diseases (63). Immune complex coated MPs might be more immunogenic compared to MPs without immunoglobulins, since they are more likely to deposit in the microvasculature, thereby causing inflammation and tissue damage (114). However, also *in vitro* generated MPs without autoantibodies can confer inflammatory effects on other cells (93).

### Endocytosis of MPs

The endocytosis of MPs delivers autoantigens into the endosomal compartments of the target cells. Endocytosis is a rapid (115, 116), energy dependent (117, 118) process and it is facilitated by interactions between proteins of MPs and proteins of recipient cells (117–119). A combination of multiple protein-protein interactions rather than a single protein-protein interaction seems to direct MP uptake (120, 121), which most probably contributes to the specificity of MP delivery. As mentioned earlier, tetraspanins which are highly abundant on MPs such as CD9, CD63, CD81, were shown to be involved in vesicular fusion events contributing to the targeting and uptake of MPs (82, 122–125). Similarly, integrins including ICAM-1, CD11a, CD52, and CD61 contribute to the uptake of MPs (117, 126–128). Proteoglycans such as heparan sulfate proteoglycans (118) and lectins (126, 129) also appear to be involved in MP-recipient cell interaction and MP uptake. Several endocytic pathways including phagocytosis, clathrin- and caveolin-mediated endocytosis facilitate internalization of MPs [reviewed in (130)].

Endosomes containing MPs, might ultimately fuse with lysosomes leading to degradation of the endosomal content. Within the different endosomal compartments, receptors can sense the cargo of the received MPs. Nucleic acids and modified chromatin, especially present within MPs derived from apoptotic cells stimulate pattern-recognition receptors, such as Toll-like receptors (TLRs) (37, 93). The immunogenicity of MPs containing nucleic acids has been recently demonstrated by an elegant study showing the importance of the nuclease DNase1 like-3 (DNase1L3) in the degradation of nucleic acids in MPs. Lack of Dnase1L3 results in the development of lupus-like disease in mice with the characteristic production of high anti-DNA antibody titers (10).

Depending on the type of nucleic acid, either TLR7 (sensing single strand RNA) or TLR 9 (sensing DNA) can be activated (131). TLR ligation leads to the activation of intracellular signaling pathways, thereby leading to changes in gene expression, ultimately resulting in the activation of the target cell (132). Activation of cells can either initiate the production of pro-inflammatory cytokines, trigger the increase of activation markers on the plasma membrane or induce proliferation (64). For example, dendritic cell activation by MPs leads to an increased release of interferon (IFN) type I in SLE (37, 111, 133, 134). Type I IFNs then stimulates the survival and activation of dendritic cells, B-cells and T-cells (135). MPs carrying autoantigens derived from apoptotic cells might also facilitate direct B-cell activation by engagement of the B cell receptor, together with intracellular activation of TLRs (136). Together this potentially leads to the clonal expansion of autoreactive B-cell clones and might result in the break of tolerance in autoimmune diseases.

### Antigen Presentation Facilitated by MPs

Upon internalization of MPs by antigen presenting cells, the content of MPs can be presented to T-cells. Alternatively, MPs can transport whole peptide-major histocompatibility complex (MHC) complexes from one cell to another, a process that has been termed ‘cross dressing’ (137, 138). MPs originating from apoptotic cells were shown to induce an alternative maturation of dendritic cells, characterized by down regulation of MHC II, which is in line with the immunologically silent nature of apoptosis (139). This mechanism however was found to be disturbed in patients with systemic lupus erythematosus (SLE), where apoptotic MPs were found to mature dendritic cells without MHC II down regulation (139). This has been explained by the finding that MPs originating from apoptotic cells contain a myriad of modified autoantigens, especially modified chromatin, that can trigger the activation of antigen presenting cells like dendritic cells (37, 93, 111, 140, 141). This difference in dendritic cell maturation might explain part of the autoimmune reaction observed in patients with SLE.

Antigen presenting cells themselves can also release MPs, which may activate T- and B-cells. MPs containing MHC I or MHC II complexes loaded with antigens, together with co-stimulatory molecules can augment the proliferation of specific T-cells as has been shown for dendritic cell derived exosomes (127, 142, 143).

## The Deleterious Properties of MPs in Disease

It has become clear that MPs have potent pro-inflammatory effects, however they also can stimulate coagulation and can affect vascular function (144, 145). Owing to this, MPs have been assigned deleterious roles in a plethora of pathologies, predominantly cardiovascular and autoimmune diseases (146). In this and the following section, a brief overview on the role of MPs in various clinical settings is provided.

### MPs Are Pro-Coagulant

Exposure of negatively charged PS on apoptotic MPs facilitates the binding of coagulation factors to these MPs and the assembly of coagulation enzyme complexes (147). Coagulation factors become readily activated on MPs as they co-express high levels of membrane-anchored tissue factor (148). Indeed, MPs isolated from cell lines or human plasma readily support thrombin production *in vitro* (65, 149, 150). Large von Willebrand factor multimers have been detected on endothelial cell-derived MPs, suggesting that MPs can also facilitate the aggregation of platelets during coagulation (39, 50). Besides direct pro-coagulant properties of MPs, they may also indirectly facilitate coagulation. For instance, leukocyte-derived MPs have been shown to contain tissue factor and P-selectin glycoprotein ligand-1 (PSGL-1). Those MPs accumulate at thrombi rich in platelets *via* the interaction of platelet P-selectin and PSGL-1, bringing tissue factor to the growing thrombus (151).

### MPs Cause Endothelial Dysfunction

Experiments with MPs isolated from the blood of women with pre-eclampsia or patients with myocardial infarction showed that MPs can alter vascular function. Notably, these MPs were endowed with the capacity to diminish endothelium-dependent relaxation of arteries *ex vivo* (152, 153). MPs isolated from healthy donors have been shown to harbor endothelial nitric oxide synthase (eNOS) required for vascular homeostasis, and eNOS-containing MPs appear to be specifically decreased in patients with endothelial dysfunction (154). MPs can also scavenge nitric oxide and thereby cause endothelial dysfunction (155, 156). Furthermore, MP-induced increase in superoxide production in endothelial cells can decrease endothelium-dependent relaxation (157).

## MPs in Autoimmune Diseases

Under normal conditions, MPs derived from platelets or megakaryocytes are the most common subpopulation, comprising around 70% of all MPs in the circulation (158). MPs are released in healthy individuals under basal conditions. Various clinical conditions have been associated with alterations in MP counts or changes in specific MP subpopulations. Nevertheless, it is still uncertain whether changes in quantity or characteristics of MPs in autoimmune diseases are the cause or consequence of disease. Although an increased presence of MPs could be explained by disease related pathogenic events, the direct effects of MPs on coagulation, inflammation and endothelial dysfunction strongly suggest that MPs can also be involved in the etiology of the disease. Supporting such a causal role, Sisirak and colleagues showed that a mouse model deficient in DNase1L3 displayed an increased presence of chromatin in MPs, thereby leading to the onset of autoimmunity (10). Owing to their procoagulant and proinflammatory properties, it is not surprising that MP alterations are predominantly connected to cardiovascular and autoimmune diseases. MP alterations in the context of cardiovascular disease is beyond the scope of this review and has been extensively reviewed elsewhere (6, 7, 159, 160).

### Antiphospholipid Syndrome

Antiphospholipid syndrome (APS) is an autoimmune disease that clinically manifests as recurrent thrombotic events and pregnancy-related issues such as stillbirths and (pre)-eclampsia. Laboratory abnormalities include increased levels of autoantibodies against membrane anionic phospholipids, such as anti-cardiolipin and anti-PS autoantibodies. APS can occur in association with other rheumatic disorders, such as SLE, but can also be a clinical entity on its own. The pathogenesis of APS remains poorly understood, but MPs could explain the procoagulant and proinflammatory phenotype of APS (161). Indeed, high levels of endothelial cell-derived tissue factor-expressing MPs have been identified in APS patients (162). Simultaneous analysis of primary APS patients and SLE patients with and without APS showed that especially primary APS patients as well as SLE patients with APS displayed elevated levels of endothelial cell-derived MPs, in contrast to SLE patients without APS (163). *Ex vivo* experiments with plasma from patients with various autoimmune diseases demonstrated that in particular APS plasma induced endothelial-derived MPs with procoagulant properties (163). Separate analyses of APS patients with only obstetric or only thrombotic complications revealed that endothelial cell-derived MPs are increased in thrombotic but not in obstetric APS patients (48). In addition to endothelial cell-derived MPs, elevated levels of platelet-derived MPs have also been reported (48, 164). Collectively, these data suggest a link between anti-phospholipid antibodies, endothelial cell-derived MPs and the development of thrombosis in APS.

### Systemic Sclerosis

Systemic sclerosis (SSc) is characterized by degenerative changes, diffuse fibrosis and vascular abnormalities in various organs. Raynaud phenomenon, dysphagia, skin tightening and polyarthralgia are amongst its symptoms. Studies report different MP numbers in patients with SSc. Some studies show elevated numbers of MPs (70, 158, 165, 166), whereas other studies report lower MP counts in patients with SSc (40, 60). Nevertheless, MPs have been associated with certain clinical manifestations, such as interstitial pneumonia or perivascular inflammation (40, 165), whereas MP numbers were inversely correlated with skin thickness scores (158). Maugeri et al. showed that platelet derived MPs contain the damage associated molecular pattern high mobility group box 1 (HMGB1), which is able to induce the formation of NETs by neutrophils (70). One study assigned MPs as active players in the pathogenesis of SSc. The inhibition of MP release by endothelial cells using pantethine or by inactivating ATP-binding cassette transporter A1 (ABCA1), thereby inhibiting transport of PS to the outer leaflet, ameliorated skin and lung fibrosis in murine SSc (167). Notably, pantethine had earlier been shown to protect against cerebral malaria by impairing MP release from activated endothelial cells (168). Altogether, these data suggest that MPs play a role in the disease development of SSc.

### Primary Sjögren’s Syndrome

Primary Sjögren’s syndrome (pSS) is a chronic autoimmune disease in which fluid-secreting glands become inflamed. This primarily results in the development of a dry mouth and dry eyes. Other symptoms can include fatigue, muscle and joint pain and a chronic cough. Genetic, hormonal and environmental factors culminate in the infiltration of lymphocytes into the salivary and lacrimal glands, thereby causing their dysfunction. Increased numbers of endothelial cell-derived MPs have been reported for pSS patients and are considered to reflect the degree of systemic endothelial damage (41). Levels of endothelial cell-derived MPs directly correlate with disease duration (41). In a comparative analysis of patients with pSS, SLE and RA, it was found that all patient cohorts had increased levels of platelet-derived MPs, but that increased levels of leukocyte-derived MPs were exclusive for pSS (169). Exosomes released from salivary gland epithelial cells contain the typical autoantigens that are targeted in pSS, i.e. Ro/SSA, La/SSB and Sm ribonucleoproteins (RNPs), thereby suggesting that the release of such exosomes directly contributes to the autoimmune response in pSS (170). In summary, there is a wide spectrum of elevated MP populations in pSS, that influence key events in its pathogenesis, such as platelet activation, systemic endothelial damage and injury to salivary gland epithelial cells.

### Rheumatoid Arthritis

Rheumatoid arthritis (RA) is an inflammatory autoimmune disease causing chronic joint inflammation and systemic manifestations, including accelerated forms of cardiovascular disease. Multiple factors, such as genetic background, epigenetic changes and environmental triggers culminate in the production of autoantibodies, predominantly against citrullinated proteins that cause joint inflammation and subsequent bone destruction. MPs are perceived as important mediators of RA disease, both locally in the joints as well as systemically to promote vascular alterations. In particular, elevated levels of platelet-derived MPs have been identified in RA and MP levels correlate with disease activity (71, 171, 172). Platelet-derived MPs are also discovered in synovial fluid of RA patients, in addition to their presence in the circulation (64). Another study claims that MPs derived from granulocytes and monocytes are predominantly found in synovial fluid of RA patients, whereas there was no increase of MPs derived from platelets (173). This discrepancy is most likely explained by the use of different markers to define the origin of MPs. It appears that particularly platelet-derived MPs are active players during inflammatory arthritis, as the depletion of platelets drastically attenuates the severity of arthritis in mouse models (64). Furthermore, MPs isolated from RA joints are capable of activating synovial fibroblasts *in vitro* (174). Notably, MP-induced fibroblast activation causes the secretion of matrix metalloproteinases and the production of pro-inflammatory cytokines (9). Additionally, MPs in RA patients containing autoantigens can form immune complexes leading to inflammation in the circulation and joints (175). Matrix metalloproteinases degrade extracellular matrices and consequently cause cartilage and bone destruction. Altogether, it appears that there is an important role for MPs, in fueling the local and systemic inflammatory response in RA patients.

### Vasculitis

Endothelial injury is central in the pathogenesis of vasculitis and is associated with elevated levels of endothelial cell-derived MPs and platelet-derived MPs (176, 177). Endothelial cell-derived MPs are particularly elevated in patients with active disease compared to those in full or partial remission (178). Importantly, levels of endothelial cell-derived MPs correlate with disease activity scores and acute-phase markers (179). In children with vasculitis, platelet-derived MPs are also elevated, but to a lesser extent than those derived from endothelial cells (179). Neutrophil-derived MPs are also reported in vasculitis patients with anti-neutrophil cytoplasmic antibodies (ANCA). Polyclonal ANCA as well as chimeric proteinase 3 (PR3)-ANCA can stimulate neutrophils to release MPs, which in turn express PR3 and myeloperoxidase and bind to endothelial cells in a CD18-dependent manner (51). The latter results in the expression of endothelial adhesion molecules and the secretion of pro-inflammatory cytokines. Thus, neutrophil-derived MPs may connect ANCA with vascular damage in patients with ANCA-associated vasculitis.

### Systemic Lupus Erythematosus

SLE is a devastating disease in which autoantibodies develop against nuclear components, such as anti-DNA, anti-histone and anti-RNP autoantibodies. The binding of anti-nuclear autoantibodies to their antigens results in the formation of immune complexes, which deposit in various organs, thereby causing inflammatory tissue destruction due to complement activation and leukocyte recruitment. MPs are perceived as important reservoirs of autoantigens in SLE, as outlined above (37, 109, 180, 181). Central to the pathogenesis of SLE is the failure to adequately clear apoptotic cells and apoptotic MPs (182–184). There are conflicting reports about MP counts in SLE patients compared to healthy controls, ranging from increased numbers of MPs, or no differences to even reduced numbers of MPs (37, 52, 55, 59, 61, 62, 71, 93, 110, 113, 114, 169). A fundamental difference between endothelial cell-derived MPs in SLE versus MPs in RA and SSc is that chromatin is solely present in MPs from SLE patients (37). Chromatin within SLE MPs contains specific modifications to N-terminal histone tails that are associated with apoptosis, as outlined above (140, 185, 186). In contrast, endothelial cell-derived MPs in RA and SSc patients are more likely to reflect endothelial cell activation. As outlined, proteomic analyses of MPs confirmed that apoptotic proteins are specifically present in SLE MPs but not in those from patients with other rheumatic diseases (78, 187). Although MP levels do not seem to directly correlate with disease activity scores, MPs rich in apoptosis-modified chromatin appear to be specifically present in SLE patients with lupus nephritis (93). In summary, SLE MPs are unique compared to MPs in other autoimmune diseases in that they contain high levels of apoptosis-modified chromatin, thereby providing these MPs with potent proinflammatory properties that may enhance the inflammatory response in SLE.

## Concluding Remarks

MPs are important players in normal homeostasis as well as in pathological conditions. MPs can serve as mediators of long-distance communication between cells and deliver different kind of cargoes or signals throughout the body. The study of MPs isolated from body fluids, especially plasma, remains challenging since there is no consensus on protocols of how to isolate and analyze number and origin of MPs. To improve comparability between different studies, the International Society for Extracellular Vesicles published consensus papers with guidelines addressing many important issues concerning MP research (26, 36). For the time being differential centrifugation and flow cytometric analyses are the techniques of choice for most researchers in the field. However, new and exciting developments, especially the isolation of MPs by microfluidics and characterization of MPs with mass spectrometry, may change the way of how to isolate and analyze MPs in the future.

MPs released from activated or dying cells can have a pathogenic role in numerous autoimmune diseases by fueling inflammatory processes, coagulation and endothelial dysfunction. Nevertheless, the scientific evidence connecting MPs to autoimmune diseases is predominantly associative in nature and fails to unambiguously answer the question whether MPs are the cause or consequence of disease. To solve this issue, a more thorough understanding of why and how MPs are produced in different disease settings is needed. Additionally, strategies to control the release or the clearance of MPs are needed to investigate the effects of increasing or decreasing MP concentration in health and disease. Alternatively, exogenously administered vesicles could be used to proof that vesicles are causing autoimmunity. Exogenous administration of MPs comes with its own challenges as the source of MPs, as well as the route and dose of administration impacts the final biodistribution (188). Nevertheless, infusion of extracellular vesicles have pathologic effects in different mouse models of preeclampsia or multiple sclerosis (189, 190). In conclusion, findings obtained from research focusing on casual relations rather than associations will facilitate the development of disease-specific therapeutic strategies that can regulate MP release or, alternatively, ensure their immunologically silent clearance; opening new avenues for the treatment of autoimmune diseases in which MPs play a causative role.

## Author Contributions

NR, CY, and EP wrote the manuscript. NR and CY prepared the figures. LH and JvdV contributed to writing and critically revised the manuscript. All authors contributed to the article and approved the submitted version.

## Funding

This research was supported by the Radboud Institute for Molecular Life Science (RIMLS) PhD program.

## Conflict of Interest

The authors declare that the research was conducted in the absence of any commercial or financial relationships that could be construed as a potential conflict of interest.

## Publisher’s Note

All claims expressed in this article are solely those of the authors and do not necessarily represent those of their affiliated organizations, or those of the publisher, the editors and the reviewers. Any product that may be evaluated in this article, or claim that may be made by its manufacturer, is not guaranteed or endorsed by the publisher.
